# Assessment of Directional–Hemispherical Reflectance of Tablets with Cefuroxime during Storage under Elevated Temperature and Ultraviolet Radiation

**DOI:** 10.3390/s24020630

**Published:** 2024-01-19

**Authors:** Michał Meisner, Beata Sarecka-Hujar

**Affiliations:** 1 Doctoral School, Faculty of Pharmaceutical Sciences in Sosnowiec, Medical University of Silesia in Katowice, 41-200 Sosnowiec, Poland; 2 Department of Basic Biomedical Science, Faculty of Pharmaceutical Sciences in Sosnowiec, Medical University of Silesia in Katowice, 41-200 Sosnowiec, Poland

**Keywords:** hemispherical reflectance, storage, tablets, stressful conditions, stability

## Abstract

Environmental conditions can lead to changes in the physical and chemical structures of drug products. In this study, the stability of cefuroxime tablets stored under adverse conditions was evaluated based on total directional–hemispherical reflectance (THR). The THR value was measured before and after the tablets’ exposure to stress factors (temperature of 45 °C and UV radiation). Each measurement was performed three times within seven spectral bands at the beginning of the experiment (day 0), and then on days 1, 2, 3, 5, and 7. In addition, hyperspectral profiles (400–1030 nm) were analyzed on days 0 and 7. A significant decrease in THR values in all wavelength ranges was observed when day 7 vs. day 0 were compared, especially for spectral bands of 335–380 nm and 1700–2500 nm (Δ = 0.220, *p* < 0.001 and Δ = 0.171, *p* < 0.001, respectively). The hyperspectral analysis confirmed a decrease in the reflectance after the end of stress conditions in the visible light range (400–700 nm) compared to tablets before the experiment. This may indicate that more radiation entered the tablets. In conclusion, the THR of cefuroxime tablets decreases during the exposure to heat and UV radiation, which may result from some physicochemical changes that have occurred during storage.

## 1. Introduction

Light incident on an opaque boundary surface undergoes partial absorption and partial reflection. This reflection, in its extreme form, can be either specular reflection (when the laws of reflection are met) or diffuse scattering [1]. Directional–hemispherical reflectance is the ratio of the light radiation flux reflected by a unit of the surface into the view hemisphere to the illumination radiation flux when the surface is illuminated by a parallel beam of light falling from one direction [2]. The method has been widely used for years in scientific fields including geology, the food industry, as well as military or space research. In recent years, the hemispherical reflectance technique has also been used in biomedical and pharmaceutical analyses [3,4,5].

Technologies for drug development and control are constantly evolving and different devices are available to measure radiation reflected from the surface. Among these instruments, a portable apparatus for the rapid acquisition of directional observations of land and atmosphere has been used recently [6]. This compact device enables the measurement of hemispherical reflectance of various surfaces within a wide range of spectral bands. 

The shelf life of a solid drug formulation is one of the key characteristics of a finished drug product. This property describes the length of time a drug product will retain its ability to exert its intended therapeutic effect and also ensures the safety of the drug’s use during this period. Many patients store medications before use contrary to the manufacturers’ recommendations [7]. In consequence, environmental conditions such as elevated temperature or UV radiation [8,9] may lead to the loss of original properties. For this reason, it is extremely important to properly protect the finished product and store it under appropriate conditions. Due to the variability of each batch, the stability and quality of extended-release drug products can only be ensured through periodic testing and systematic evaluation of each batch [10]. Adequate control of medical products contributes to improving the efficacy of ongoing pharmacotherapy; for this reason, it is extremely important to provide appropriate tools for rapid screening inspection of finished preparations.

Many studies have demonstrated the influence of light energy on the photostability of various active pharmaceutical substances (APIs) and finished pharmaceutical products [11,12]. Also, the mechanisms of photodegradation of many APIs are known [13,14]. However, scarce data show the effect of particular spectral ranges of radiation on pharmaceutical formulations. For the most part, these are long-term tests and involve the destruction of samples.

Because the surfaces of different substances absorb, transmit, or reflect radiation to different degrees, it is possible to use reflectance measurements to assess the shelf life of a solid form of a drug. Under the influence of external factors, both chemical and physical changes can occur in the stored product. Both types of the aforementioned changes can induce a change in the reflectance of the test sample. Due to the ease and speed of the measurements performed, the measurement of directional–hemispherical reflectance is an interesting and modern alternative to conventional methods of assessing the quality of pharmaceutical preparations.

The purpose of this study was to evaluate for the first time to the authors’ knowledge the shelf life of tablets containing cefuroxime during storage under adverse conditions based on the total directional–hemispherical reflectance (THR) measured. The main assumption of the research was that there would be a change in the value of THR of the tested solid drug forms during several days of exposure to elevated temperature and UV radiation.

## 2. Materials and Methods

### 2.1. Pharmaceutical Preparation

In this study, coated tablets containing cefuroxime (500 mg; expiration date October 2025) were analyzed. According to the characteristics of the medicinal product, excipients used for the preparation of the core of cefuroxime tablets were microcrystalline cellulose, sodium lauryl sulfate, croscarmellose sodium, hydrogenated vegetable oil, and colloidal anhydrous silica [15]. The tablet coating consisted of hypromellose, propylene glycol, methyl parahydroxybenzoate, propyl parahydroxybenzoate, and white opaspray (containing, among others, titanium dioxide and sodium benzoate) [15]. A visual evaluation of the analyzed tablets was conducted at the beginning of the study (day 0) and after seven days of storage under stressful conditions, and the external appearance was assessed under both visible and UV light using an Olympus Tough camera with a Dermlite attachment (Olympus Europa Se & Co. KG, Hamburg, Germany).

### 2.2. Research Procedure

Tablets with cefuroxime were stored under stressful conditions at an elevated temperature of 45 °C and under the influence of radiation for 7 days in an aging chamber (Solarbox 1500, Cofomegra.srl, Milan, Italy). A xenon lamp, which is installed in the chamber, emitted the entire spectrum of radiation with an irradiance range from 250 to 1.100 W/m^2^. In our experiment, we used a UV filter (S206/S406), which is installed between the lamp and the chamber and modifies the xenon spectral curve in the UV area. This is a soda lime glass filter with an extra-long life for indoor exposure, i.e., it simulates solar radiation filtered through window glass.

All analyzed tablets (*n* = 10) were assessed in terms of hemispherical–directional reflectance on subsequent days of the experiment, i.e., before beginning the experiment (day 0) and then on days 1, 2, 3, 5, and 7. In addition, we analyzed the hyperspectral profiles of the tablets before beginning the experiment (day 0) as well as at the end of the experiment (day 7).

#### 2.2.1. Hemispherical–Directional Reflectance

The measurements of hemispherical–directional reflectance were performed with a 410-Solar Reflectometer (Surface Optics Corporation, San Diego, CA, USA). The device measures total, diffuse, and specular reflectance; however, we focused our attention only on total hemispherical reflectance (THR). The THR measurement is taken within seven wavelength bands from 335 nm to 2500 nm at an angle of 20°. Thus, the beam includes ultraviolet, visible, and near-infrared light. There are two main elements in the structure of the SOC410 series reflectometer: an optical measuring head with an integrated light source, which is a tungsten filament, and a battery-powered control module. This makes the reflectometer portable for measurements in the field and/or during the manufacturing process. In turn, the remote control unit is suitable for laboratory and stationary measurements, where the device is powered by an outlet. The integrating sphere captures the reflected radiation from the tested object, integrating reflections in all directions. Each of the selected tablets was measured three times on each measurement day, which gave a total of 30 measurements in a given time interval. The spectral data were obtained using the solar energy distributions for air mass 1.5, which corresponds to a solar zenith angle of 48°.

#### 2.2.2. Hyperspectral Imaging

A hyperspectral analysis was performed using a Specim IQ hyperspectral camera (Spectral Imaging Ltd., Oulu, Finland). The device allows for the acquisition of images within the wavelength range of 400–1000 nm and a spectral resolution of 5 nm. The measurements were made every 3 nm, i.e., across 204 spectral bands. The spatial resolution was 512 × 512 with a pixel size of 17.58 μm × 17.58 μm. The distance from which the images were recorded with a focal length of 21 mm was 30 cm. A white reference was used for each measurement to measure incident light seen by the camera before it reached the test sample. The images of the tablets were recorded using an optimized external lighting system including two incandescent lamps with flat spectral characteristics within the spectral range of the device, i.e., 400–1000 nm. For the applied lighting system, the integration time for a single frame was from 12 ms to 19 ms. The analyses of images, conversion of raw data into a matrix, as well as extraction of the selected features were performed using MATLAB version 7.11.0.584 (R2010b) software.

The maximum, average, and minimum values of reflectance over the entire surface of the tablet were evaluated.

### 2.3. Statistical Analysis

Statistical analysis of the obtained data was performed using Statistica 13 (StatSoft, Tulsa, OK, USA) and Microsoft Excel 2019 (Office 365, Microsoft Corporation, Redmond, WA, USA). Data were presented as median (min.–max.) or mean ± standard deviation (M ± SD). The Shapiro–Wilk W test was used to assess the normality of data distributions. If the distribution of a given variable in the compared groups was normal, Student’s *t*-test was used; for data deviating from the normal distribution, the nonparametric Mann–Whitney U test was used.

To determine the relationship between more than two groups, the Kruskal–Wallis non-parametric ANOVA test was used. If the result of this test was significant, post hoc analyses were performed using the Bonferroni test to assess the differences between individual pairs of preparations.

Time-dependent continuous variables (THR measurement before the experiment (day 0) and then on days 1, 2, 3, 5, and 7) were analyzed using repeated-measures ANOVA. If a statistical relationship between three measurements was found, the Bonferroni post hoc test was used to compare pairs of measurements. A *p*-value less than 0.05 was considered a significant result.

## 3. Results

### 3.1. Characteristics of Tablets

**Figure 1:**
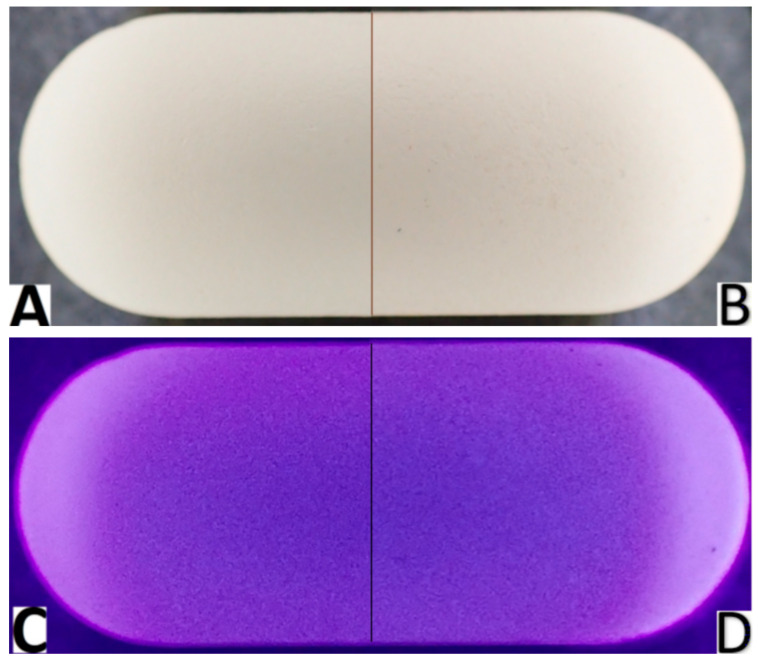
Images comparing visible appearance of the analyzed tablets with cefuroxime at the beginning of the study (day 0) under visible light (**A**) and under UV light (**C**) and on 7th day of the experiment (day 7) under visible light (**B**) and under UV light (**D**). In the tablets after 7 days under stress conditions, disruptions in the homology of the internal structure were visible.

The analyzed tablets are white, capsule-shaped, smooth on one side, and with an embossed inscription on the other side. On the seventh day of the study, compared to the beginning of the study, visual changes could be observed on the surface of the tablets, including a slight change in color shade, and more small, barely visible spaces, suggesting a break in the continuity of the surface (Figure 1). These changes could be better observed under UV light (Figure 1D vs. Figure 1C, respectively).

### 3.2. Analysis of THR Values during the Experiment

The lowest median values of THR at the beginning of the experiment (day 0) were observed within the range of 335–380 nm. For the remaining wavelength ranges, the highest median THR was found within the ranges of 590–720 nm and 700–1100 nm. Table 1 presents the exact median (min.–max.) values of THR on days 0, 1, 2, 3, 5, and 7 within all spectral bands.

The ANOVA with repeated measures showed significant differences in changes in THR in all spectral ranges between the study days (Table 1).

**Figure 2:**
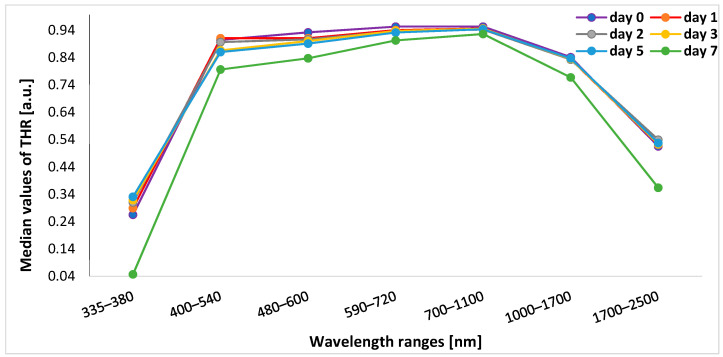
Graphs presenting median values of THR in days of subsequent measurements. THR—total hemispherical reflectance.

In post hoc analysis, in the range of 335–380 nm, THR values on a given day of the study differed significantly from THR values on subsequent days (*p* < 0.001). In the range of 400–540 nm, the value of THR on day 0 significantly differed from THR values on other days (i.e., days 1, 2, 3, 5, and 7) (*p* < 0.001), and THR values on day 7 were significantly different from THR values on each other day (*p* < 0.001). In turn, for 480–600 nm, THR values on days 0, 7, and 5 differed significantly from THR values on subsequent days (*p* < 0.001) except for day 5 vs. day 4 (*p* = 0.037). Within the range of 590–700 nm, THR values on days 0 and 7 differed from other measurements (*p* < 0.001) and day 3 vs. day 5 (*p* = 0.045). For the 700–1100 nm and 1000–1700 nm bands, THR values on days 0 and 7 differed significantly from THR values on subsequent days (*p* < 0.001). In the 1700–2500 nm range, THR values on days 0 and 7 were different from values obtained on other days of the experiment (*p* < 0.001), and in addition, day 2 vs. day 3 (*p* < 0.001), day 3 vs. day 4 (*p* = 0.002), day 3 vs. day 5 (*p* < 0.001), and day 4 vs. day 5 (*p* = 0.007) (Figure 2).

### 3.3. Analysis of Change in THR Value during the Experiment Depending on the Spectral Range

**Figure 3:**
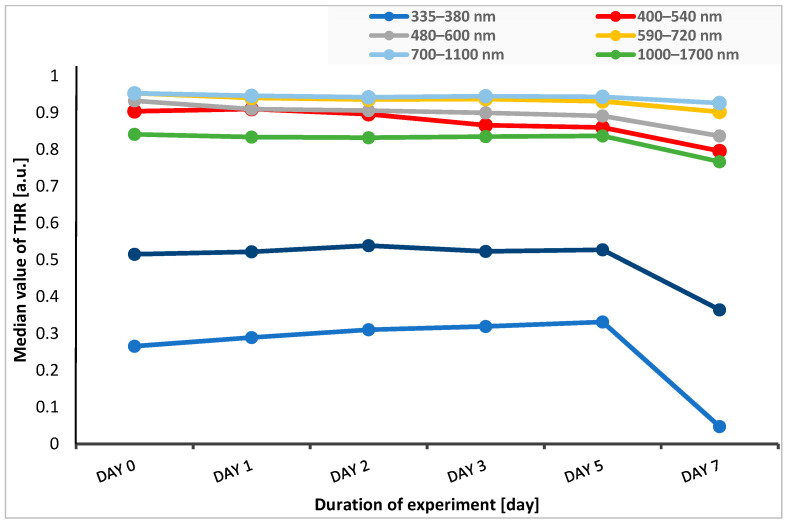
Change in the median THR over time of experiment for each spectral band. THR—total hemispherical reflectance.

In five spectral bands of 400–540 nm, 480–600 nm, 590–720 nm, 700–1100 nm, and 1000–1700 nm, the median THR values were at a similar level or slightly decreased from the time before the start of the experiment until the fifth day after application of stressful conditions (heat, UV radiation). In the case of reflectance measured in marginal ranges, i.e., 335–380 nm (UV light) and 1700–2500 nm (near-infrared light range), we observed an increase in reflectance until the second day of the experiment for the 1700–2500 nm range, while for the 335–380 nm range, there was an increase in reflectance until the fifth day of this study. Then, on the seventh day of the experiment, the reflectance values significantly decreased (Figure 3).

**Figure 4:**
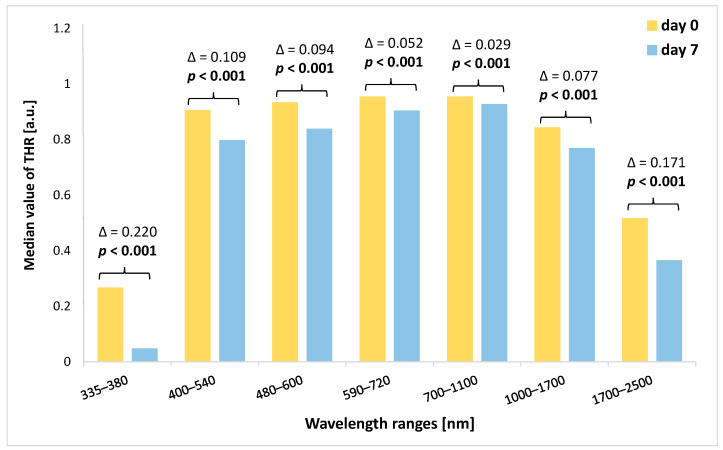
Comparison of THR values at the beginning of the study (day 0) and on the seventh day of the study for each analyzed spectral band together with the value of Δ. THR—total hemispherical reflectance; Δ—difference between the value of THR at the beginning of the study (day 0) and the value of THR on day 7. Significant differences are in bold.

When we analyzed the amount of change in the THR value (the THR value on day 7 was subtracted from the THR value on day 0 of the experiment), we noted that the highest decrease in THR characterized the 335–380 nm and 1700–2500 nm ranges (Figure 4). In turn, the lowest decrease in median values of THR was found within the 700–1100 nm spectral band. However, in all spectral bands, the differences in THR values between the beginning of the study and THR values on the seventh day of the experiment were significant (*p* < 0.001). 

### 3.4. Hyperspectral Imaging

**Figure 5:**
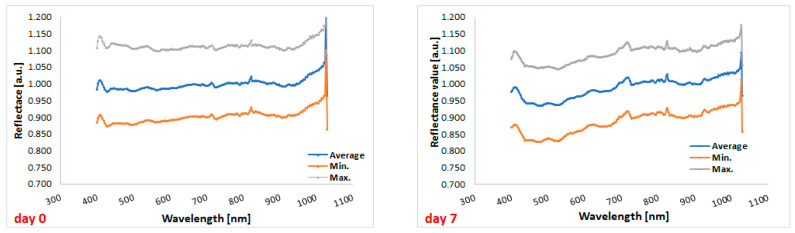
Hyperspectral profiles of the tested tablets at the beginning of the study (on the **left**) and at the end of the study (on the **right**). Gray, blue, and orange lines correspond to the maximum, average, and minimum reflectance, respectively.

Comparison between the hyperspectral profiles of cefuroxime tablets before the start of the experiment (day 0) and the profiles of the cefuroxime tablets on day 7 (i.e., at the end of the experiment) indicated that storage under stressful conditions has an impact on the reflectance of the tablets (Figure 5).

**Figure 6:**
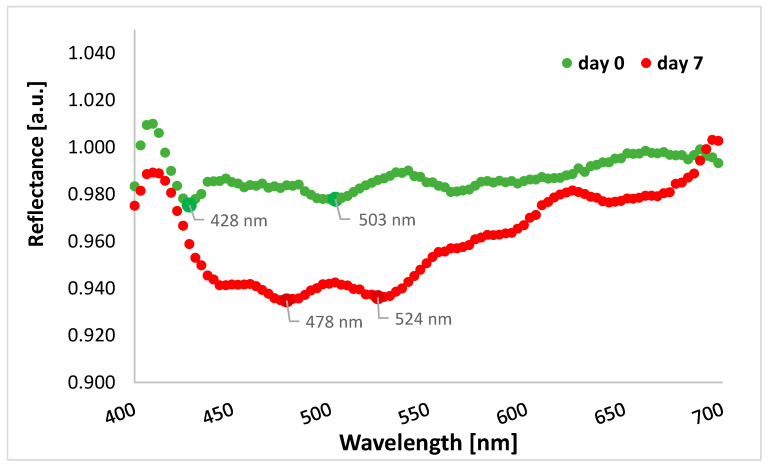
The average reflectance of the tablets with cefuroxime before starting the experiment (day 0, green curve) and at the end of the experiment (day 7, red curve) for the spectral range of 400–700 nm (visible light band).

The graph shows lower reflectance for cefuroxime tablets after the end of stressful conditions in the range from 400 to 700 nm, i.e., in the visible light range, compared to tablets before the experiment (Figure 6). Therefore, overall, in this spectral range, more radiation penetrates the tablets and may cause physical changes in the formulation. In the graph below, there are two lowest points for tablets’ reflectance measured on day 0, i.e., 428 nm and 503 nm (reflectance values of 0.975 and 0.978, respectively), as well as two lowest points for tablets’ reflectance measured on day 7, i.e., 478 nm and 524 nm (reflectance values of 0.935 and 0.936, respectively).

## 4. Discussion

In the present study, the possibility of reflectance measurement in regard to assessing the shelf life of a solid pharmaceutical preparation was tested. The assumption of the research was that using a directional–hemispherical reflectometer may offer a non-destructive, non-invasive, rapid, and time-saving method to obtain new knowledge on pharmaceutical stability. For this purpose, the total hemispherical reflectance (THR) was measured for tablets containing cefuroxime before, during, as well as after exposing them to stress factors, i.e., elevated temperature of 45 °C and UV radiation. The device used analyzes reflectance in seven discrete spectral bands, from UV ranges through visible light to infrared. The results obtained from the experiment show a significant decrease in the values of THR in all tested wavelength ranges after 7 days of stressful conditions.

In this study, the temperature of 45 °C was chosen to accelerate the possible physical changes that may occur during drug storage. Previously, it was demonstrated that almost one-third of patients from China did not pay attention to temperature during drug storage [16]. An elevated temperature is feasible in some places in the house where, especially older patients, may temporarily store medications. In the study by Vieland et al. [17], most of the surveyed older patients stored their medicines in the kitchen, with a mean temperature of 20.2 °C and a maximum temperature of 30.3 °C. The authors found that the highest mean temperature was in the living room or hallway, with maxima of 27.1 °C and 33.9 °C, respectively [17]. In turn, McLean et al. [18] stored griseofulvin tablets in different temperatures of 37 °C, 50 °C, and 60 °C for 1, 2, or 4 weeks to analyze the dissolution performance after storage. However, in studies analyzing problems with drug storage, light exposure is usually not measured and the temperature, e.g., in front of the window is always higher compared to other places.

The change in the reflectance value noted in this experiment may have resulted from changes occurring on the surface of the tested formulation. The tablets used are coated and some physical changes may concern the coating itself, e.g., a change in thickness. Data on the effect of storage conditions of pharmaceuticals on the coating thickness are scarce. Previously, Zhang et al. [19] analyzed the usage of terahertz pulsed imaging (TPI) in the analysis of the structure of a tablet coating before and after an accelerated aging process for four weeks. According to the obtained results, the authors found the coating reduced from 50 µm to 40 µm in thickness and exhibited a higher density after being stored. At the same time, there was no significant effect of physical changes in the coating of the stressed tablet on the drug release efficiency. In addition to changes in the coating, there may also be changes in the chemical composition of the drug during storage. The study by Ramos et al. [20] demonstrated that UVC radiation increased the ability of the model substances with ketoconazole and miconazole to reduce free DPPH radicals, which could indicate photolysis of these APIs. In the case of our experiment, we also treated the preparations with UV radiation under the influence of which the breakdown of the active substance can occur, which could lead to changes in reflectance values. Previously, it was observed that among antibiotics bearing the alkoxyimino group, cefuroxime axetil was the most sensitive under irradiation at 254 nm [21]. In turn, Jiang et al. [22] conducted a study on the degradation of four cephalosporins in an aqueous environment. The half-lives of all cephalosporins decreased significantly after exposure to sunlight compared to substances stored in dark conditions.

In the present study, we additionally performed a hyperspectral analysis of the cefuroxime tablets at the beginning of the experiment and after the whole experiment to confirm the change in the reflectance profile. The camera used in the experiment measured reflectance within visible light and infrared, i.e., from 400 nm to 1030 nm; therefore, the analyzed wavelength range was narrower than that of the reflectometer. Within the wavelengths of visible bands, we observed significant differences in values of reflectance between the beginning and the end of the experiment. This means more light was transferred to the tablets after applying heat and UV radiation.

Previously, hyperspectral imaging combined with principal component analysis was used by Al Ktash et al. [23] to prepare a device to distinguish drug products by their contained drug substance on the production line. The value measured by the apparatus built was the reflectance, on the basis of which the active substance was evaluated. The authors found the method appropriate to distinguish the tested preparations from one another. In the course of experiments, we also used the hyperspectral imaging method, whose results coincided with those obtained with the reflectometer. In earlier studies by other researchers, it was proven that reflectance measurement makes it possible to distinguish between a genuine and a counterfeit drug [3]. In this case, the method distinguished between the compared preparations due to differences resulting from different methods of production of the medical preparations in question.

As the present research showed that more UV light could penetrate the tablets during the experiment, it can be surmised that as a result of unfavorable environmental conditions, the envelope of the tested tablets degraded, which may have affected and potentially reduced the subsequent pharmacotherapy.

Despite the novelty of this research, a limitation was the lack of certain chemical analyses, as chemical changes may affect physical ones. With this in mind, in-depth research is planned to be conducted in the future.

## 5. Conclusions

The present study has demonstrated the usefulness of directional–hemispherical reflectance in the analysis of the stability of solid dosage forms. At the end of the tablets’ storage under heat and UV radiation, the value of THR decreased. Thus, in conclusion, lower hemispherical reflectance may result from physicochemical changes that occur during storage and as a consequence more light is transmitted inside the tablet.

## Figures and Tables

**Table 1:** Median values of THR measured for tablets containing cefuroxime for a particular spectral range over the whole experiment (days 0, 1, 2, 3, 5, and 7).

Spectral Ranges (nm)	Day 0	Day 1	Day 2	Day 3	Day 5	Day 7	*p* ^1^
	THR (a.u.) Median (Min.–Max.)						
335–380	0.265 (0.259–0.271)	0.289 (0.279–0.297)	0.310 (0.302–0.315)	0.319 (0.312–0.329)	0.331 (0.321–0.340)	0.047 (0.029–0.053)	**<0.001**
400–540	0.904 (0.899–0.909)	0.910 (0.871–0.918)	0.896 (0.854–0.883)	0.866 (0.849–0.883)	0.860 (0.838–0.887)	0.796 (0.766–0.824)	**<0.001**
480–600	0.932 (0.926–0.935)	0.910 (0.871–0.918)	0.906 (0.888–0.909)	0.900 (0.882–0.909)	0.891 (0.872–0.908)	0.837 (0.810–0.859)	**<0.001**
590–720	0.953 (0.946–0.956)	0.940 (0.902–0.946)	0.936 (0.922–0.944)	0.937 (0.920–0.940)	0.931 (0.916–0.941)	0.902 (0.810–0.859)	**<0.001**
700–1100	0.953 (0.947–0.956)	0.946 (0.915–0.951)	0.942 (0.932–0.951)	0.945 (0.932–0.950)	0.943 (0.929–0.949)	0.926 (0.902–0.928)	**<0.001**
1000–1700	0.841 (0.835–0.847)	0.834 (0.811–0.838)	0.832 (0.825–0.837)	0.835 (0.826–0.839)	0.837 (0.825–0.843)	0.767 (0.749–0.771)	**<0.001**
1700–2500	0.515 (0.505–0.517)	0.522 (0.513–0.529)	0.539 (0.527–0.547)	0.523 (0.508–0.527)	0.527 (0.511–0.540)	0.364 (0.360–0.379)	**<0.001**

THR—total hemispherical reflectance; ^1^ ANOVA with repeated measures; significant differences are in bold.

## Data Availability

The data presented in this study are available on request from the Department of Basic Biomedical Science, Faculty of Pharmaceutical Sciences in Sosnowiec, Medical University of Silesia in Katowice (Poland). The data are not publicly available due to privacy restrictions.
